# LINC01419 Promotes the Proliferation of Hepatoma Cells by Recruiting XRCC5 and Regulating Its Phosphorylation to Repair DNA Damage

**DOI:** 10.1155/2022/9313680

**Published:** 2022-07-19

**Authors:** Pan Liu, Xu Zhang, Qiang Fu, ChuanJiang Liu, QianKun Luo, PengFei Yu, Song Chen, HongWei Zhang, Tao Qin

**Affiliations:** ^1^Department of Hepato-Biliary-Pancreatic Surgery, People's Hospital of Zhengzhou University (Henan Provincial People's Hospital), 450000 Zhengzhou, China; ^2^Translational Research Institute Integrative Research Hub, Henan Provincial People's Hospital, 450000 Zhengzhou, China

## Abstract

**Background:**

Hepatocellular carcinoma (HCC) is one of the most common and fatal malignancies in human beings. Studies have shown that long non-coding RNAs (lncRNAs) play key parts in the occurrence and development of HCC. Although many lncRNAs have been studied in the HCC specifically for DNA damage repair, the role of LINC01419 in cellular DNA damage repair has not yet been studied.

**Objective:**

This study is aimed at exploring the biological role of LINC01419 and its potential mechanism in HCC.

**Methods:**

qRT-PCR was used to detect the expression level of LINC01419 in HCC tissues and cells, the proteins which were involved were detected by Western blot. Effect of LINC01419 knockdown on cell cycle, apoptosis, DNA damage, cell proliferation, wound healing, colony formation, and migration of HCC cells was studied in vitro.

**Results:**

The analysis showed that LINC01419 was overexpressed in HCC tissues and cells. Silencing of LINC01419 expression significantly inhibited the proliferation and migration ability of the HCC cells and resulted in cell cycle arrest at G0/G1 phase. Furthermore, the knockdown of LINC01419 increased the DNA damage, and to some extent, promoted sensitivity of HCC cells to doxorubicin. In addition, we performed RIP analysis which showed XRCC5 as a potential protein related to DNA damage repair in hepatoma cells.

**Conclusion:**

In conclusion, the LINC01419 acts as an oncogene in HCC and regulates DNA damage repair through XRCC5 in HCC cells.

## 1. Introduction

Nowadays, HCC treatments include traditional hepatectomy, liver transplant, molecular targeted, and immunity therapy [1, 2]. With the development treatment in HCC, it has improved the overall survival of HCC patients up to some extent, while long-term survival of HCC patients remains a concern [3]. The most common reasons for treatment failure are late diagnosis and lack of suitable diagnosis and treatment markers. Therefore, what needs to be addressed urgently is to make the mechanisms of HCC bring to light and develop an efficacious therapeutic target.

In terms of DNA damage, DNA double-strand breaks (DSBs) rank prominently and are the more severe type of DNA damage [4]. There are two main ways of repairing DNA damage, nonhomologous DNA end joining (NHEJ) and homologous recombination (HR); NHEJ is the major pathway for repair of ionizing radiation-induced DSBs during G0, and in the G1 phase of the cell cycle in mammalian cells, a low-precision repair method in which the broken DNA ends are joined directly without the involvement of a homologous template [5, 6]. NHEJ is comediated by two human Ku proteins, Ku70/Ku80 (XRCC6/XRCC5) and the catalytic subunit of the DNA-dependent protein kinase (DNA-PK) which bind tightly to ends of double-stranded (ds) DNA [7]. However, HR generally starts in the G2/S period, and the mechanism of HR is more complicated than that of NHEJ [8]. Poly (ADP-ribose) polymerase1 (PARP1) act as a multifunctional enzyme to catalyze many DNA repair factors involved in DNA repair process to maintain genome stability [9].

To date, long non-coding RNAs (lncRNAs) are widely involved in a variety of human diseases, including cancer, and this biological function is exerted in multiple ways. For instance, they act as signaling mediators, molecular mimetic, which supports or enhances transcription [10, 11]. Previously, researches revealed that disordered expression of lncRNA promotes HCC progression [12–14]. Although a large number of lncRNAs have been explicated, due to its noncoding role, its expression in HCC is poorly understood. It is even less known what the specific molecular mechanism of long non-coding RNAs affecting HCC is.

In our study, we first analyzed the RNA seq data obtained from TCGA for significant differences in expression of lncRNAs in HCC and normal liver tissues. Here, we found that the LINC01419 was significantly correlated with malignancy pathological parameters of patients with HCC and showed important function tumor DNA damage repair by downregulating phosphorylation of Ku80 in HCC cells. Our data points out that LINC01419 may be a valuable potential therapeutic target for the treatment of HCC, providing a new theoretical basis for the treatment of HCC.

## 2. Materials and Methods

### 2.1. Tissue Samples, Clinical Data, and Cell Culture

The surgically resected cancer tissue and matched normal liver tissue samples of 41 HCC patients were immediately stored in an ultra low temperature refrigerator or liquid nitrogen in the Henan Provincial People's Hospital. Postoperative pathologically confirmed HCC without preoperative chemoradiotherapy. We also collected clinical case data of patients for the association analysis. Informed written consents were obtained from all patients before recruiting for this study, and all the sampling and experimental procedures were supported by the Ethics Committee of People's Hospital of Zhengzhou University. The human normal liver cell line HL-7702 (LO-2) and HCC cell lines Huh-7, PLC/PRF/5, HepG2, SK-HEP-1, SMMC-7721 were purchased from Stem Cell Bank (Chinese Academy of Sciences). 1640 Medium (Biological Industries, Israel) was used to culture L-O2 and SMMC-7721 cells, and DMEM Medium (Biological Industries, Israel) was used to culture Huh-7, PLC/PRF/5, HepG2, and SK-HEP-1 cells. Fetal bovine serum was added to the above medium to prepare a medium containing 10% FBS. All cells were cultured in a humidified incubator at 37°C with a constant supply of 5% CO2 and timely passage of cells according to cell density.

### 2.2. Cell Transfection

The LINC01419-specific plasmid and negative control were constructed in the lentiviral system and transfected into HCC cells. Total RNA was extracted 48 hours after transfection and reverse transcribed into cDNA, and the transfection efficiency was detected by qPCR.

### 2.3. Quantitative Real-Time Polymerase Chain Reaction (qRT-PCR)

Total RNA extraction, reverse transcription, and qPCR were performed according to our previous studies [15], normalized with GAPDH, and calculated relative gene expression using 2^−△△Ct^ method. The primers involved in this study are listed in Table 1.

### 2.4. Cell Counting Kit-8 Assay (CCK-8)

The cells in the period of vigorous cell growth were planted in a petri dish at a density of 1 × 10^3^ cells/well, 10 *μ*L of CCK-8 enhancement solution (Beyotime Biotechnology, Shanghai, China) was added at different times,0 h, 24 h, 48 h, 72 h, and 96 h, and the absorbance values at 450 nm were measured.

### 2.5. Scratch Wound-Healing Assay

Huh-7 and PLC/PRF/5 cells were seeded in 6-well plates in advanced, after transfection the next day. Using a 200 *μ*L pipette tip to make scratches vertically at the bottom of the 6-well plate with 100% confluent HCC cells, washed with PBS 2-3 times, added medium, pictures should be taken at 0 h and 48 h in the same field, and the differences in the wound repair were calculated.

### 2.6. Colony Formation Assay

Approximately 1 × 10^3^ Huh-7 and PLC/PRF/5 cells were plated into each well of 6-well plates, and the petri dish is placed in the incubator for 14 days; the medium was regularly changed to maintain its pH and smooth growth of the cells. After 14 days, cells in the dish were fixed with paraformaldehyde, and after that, stained with crystal violet to observe the growth of cell colonies.

### 2.7. Western Blot Analysis

RIPA lysis buffer (Beijing Solarbio Science and Technology Co., Ltd., Beijing, China) was used to extract total protein from HCC cells. BCA kit (Beijing Solarbio Science and Technology Co., Ltd.) was used to detect protein concentration. Protein samples were separated by PAGE-SDS electrophoresis and then transferred to nitrocellulose filter membrane which immersion in electroporation (Beijing Solarbio Science and Technology Co., Ltd.) blocks protein bands with 5% milk for 3 h in the room temperature, incubated with primary antibody (Cell Signaling Technology, USA) overnight at 4°C, and washed the protein bands 2-3 times with 1% TBST, secondary antibody (Cell Signaling Technology), incubated for 2 h in the room temperature, then washed again with 1% TBST, and the expression of protein was detected using hypersensitivity ECL kit (Shanghai Epizyme Biomedical Technology Co., Ltd., Shanghai, China).

### 2.8. Nucleocytoplasmic Separation

The Nuclei PURE Prep Kit (Sigma-Aldrich, Darmstadt, Germany) was used to extract RNA from the nucleus and cytoplasm, collecting cells in a centrifuge tube and washed 2-3 times with PBS, digested with trypsin, and centrifuged at low speed to obtain cell pellets. RNase inhibitor was used to avoid RNA degradation. TRIzol reagent was used to extract RNA from nucleus and cytoplasm separately according to manufacturer's instructions and subsequently used for qPCR.

### 2.9. Flow Cytometry

After 48 h of HCC transfection, the cells were trypsinized and resuspended in DPBS. 75% precooled ethanol fixed the cells and washed it 3 times by DPBS before using for cell cycle analysis with Tali® Cell Cycle Solution (Thermo Fisher Scientific, Shanghai, China). The apoptosis of HCC cells were stained by Annexin V-PE/7-AAD Apoptosis Detection Kit (Vazyme Biotech Co., Ltd., Nanjing, China).

### 2.10. Comet Assay

The glass slides were soaked in 1% agarose for 2 minutes and oven-dried at 37°C. HCC cells were suspended in the 1% agarose and mixed evenly. 30 *μ*L of the cell-agarose mixtures were transferred to the glass slides to prepare a cell-containing gel layer and soaked in the lysate for 1 hour at 4°C, then incubated with alkaline electrophoresis solution for 20 minutes at 4°C, and electrophoresis was performed in alkaline electrophoresis solution for 30 minutes. Later on, slides were washed with dH_2_O, dehydrated in different concentrations of alcohol; images were taken and analyzed after staining.

### 2.11. RNA Pull-Down Assessment

Biotin-labeled LINC01419 (LINC01419-AS-probes) and its negative control (LINC01419-S-probes) (Thermo Fisher Scientific, Shanghai, China) were transfected into HCC cells for 48 h; starved cells were treated with PBS. Specific buffer (Ambion, United States) lysed cells for about 15 minutes. Coincubate M-280 streptavidin magnetic beads (Sigma-Aldrich) with cell lysate, then wash beads with yeast RNA and RNase-free BSA (Sigma-Aldrich). Western blot analysis of proteins bound to LINC01419 after TRIzol purification of RNA.

### 2.12. RNA-Binding Protein Immunoprecipitation

HCC cells in good condition were collected, and RNA-protein complex was extracted with Magna RIP RNA-Binding Protein Immunoprecipitation Kit (EMD Millipore Germany), RIP lysis buffer to extract complexes in HCC cells. Anti-XRCC5 antibody and Anti-IgG antibody were incubated with protein A/G beads for 1 h at 4°C. After that, the antibody and protein A/G bead mixture was incubated in cell lysate at 4°C for 4 hours, RNA extraction, and purification using TRIzol™ (Thermo Fisher Scientific, Shanghai, China) after centrifugation.

### 2.13. Statistical Analysis

SPSS 25.0 (Chicago, IL) statistical software was used for data analysis. Measurement data were presented in the form of mean ± standard deviation ($\bar{x}\pm s$), and the Student's *t*-test was used to compare the differences between the two groups of data. Expressed enumeration data with rate (%) and the chi-square test was used to compare the two groups.

## 3. Results

### 3.1. LINC01419 Expression in HCC Tissues and Its Association with Malignancy

**Figure 1:**
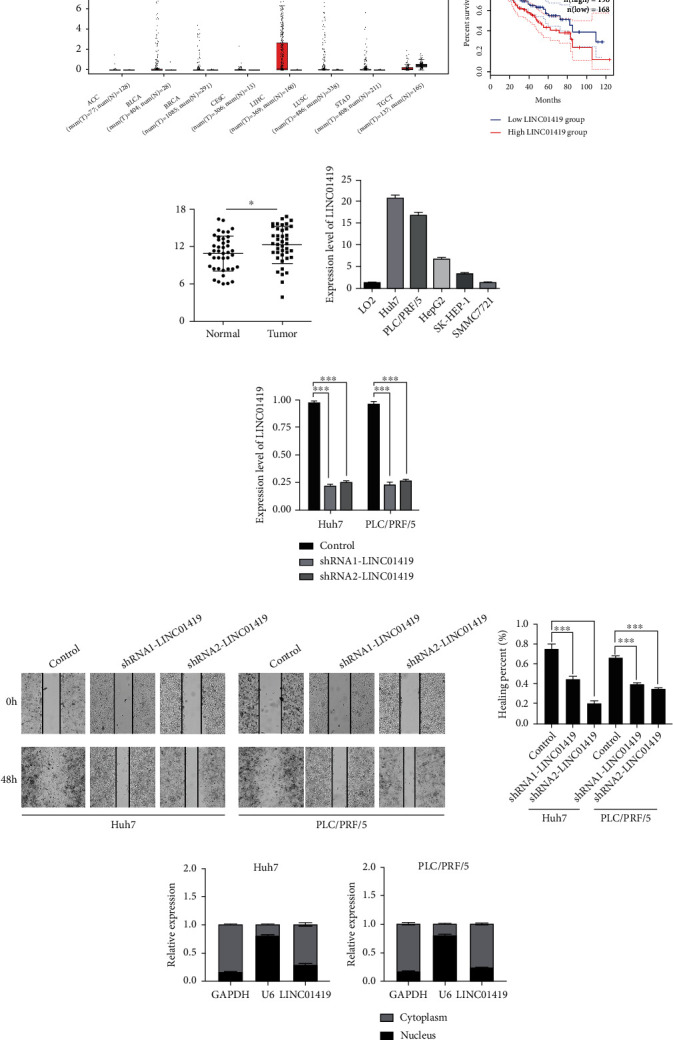
The expression level of LINC01419 is increased in hepatocellular carcinoma. (a) The expression of LINCO1419 in pan-cancer in TCGA database. (b) Elevated expression of LINC01419 is associated with poor prognosis in HCC patients. (c) The expression level of LINC01419 in tumor tissues of 41 HCC patients. (d) The expression level of LINC01419 in normal liver cell line LO2 and HCC cell lines. (e) Knockdown the LINC01419 in Huh7 and PLC/PRF/5. (f) Wound healing assay after knockdown in Huh7 and PLC/PRF/5. (g) Distribution of LINC01419 in the cytoplasm and nucleus in Huh7 and PLC/PRF/5 (^∗^*p* < 0.05, ^∗∗∗^*p* < 0.001).

In HCC and normal liver tissues, we analyzed the lncRNA expression profile. The RNA seq data of HCC and normal controls were obtained from TCGA datasets. Several lncRNAs were differentially expressed in the HCC samples, and we filtered out most significantly expressed lncRNAs, and LINC01419 was among the top in terms of significance. TCGA-HCC data from GEPIA showed that LINC01419 is highly expressed in HCC relative to other cancer types, and patients who were with high LINC01419 got worse overall survival (Figures 1(a) and 1(b)). Then, we further validated LINC01419 in clinical samples (41 HCC and paired normal liver tissues) and cell lines by using qPCR that also showed high expression level of LINC01419 in the HCC tissues (Figure 1(c)). The expression level of LINC01419 in Huh7, SMMC7721, SK-HEP-1, HepG2, and PLC/PRF/5, was also significantly higher than that in LO2 (Figure 1(d)). Furthermore, the expression level of LINC01419 was familiarly related to clinical parameters such as tumor size, tumor thrombus, and microvascular invasion in HCC patients (Table 2).

### 3.2. LINC01419 Can Promote HCC Cell Line Proliferation

**Figure 2:**
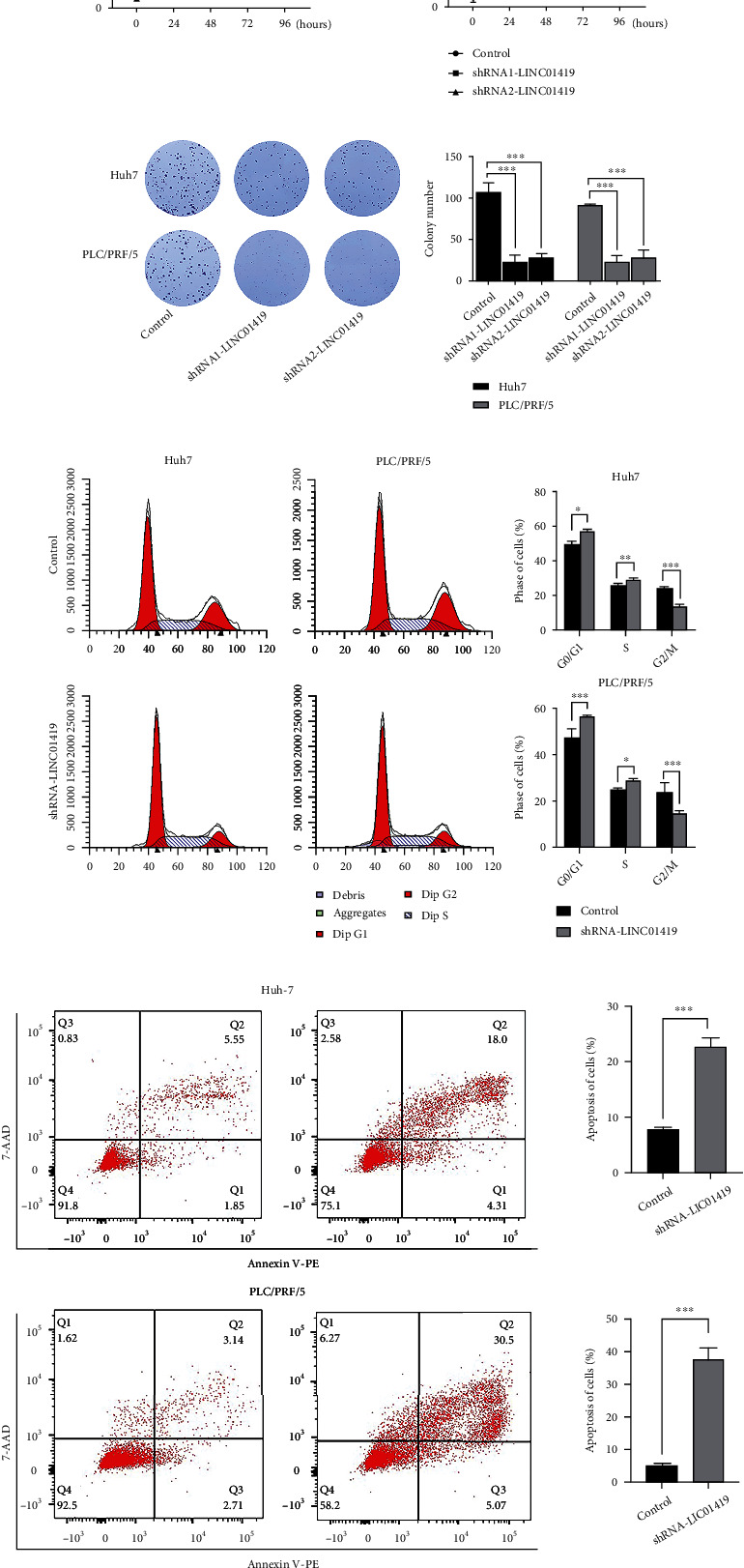
Loss of expression of LINC01419 inhibits HCC cell proliferation. (a, b) Knockdown LINC01419 inhibits the proliferation in Huh7 and PCL/PRF5. (c, d) Lack of LINC01419 causes G0/G1 phase arrested and promotes the apoptosis of in HCC cells ^(^^∗^*p* < 0.05, ^∗∗^*p* < 0.01, ^∗∗∗^*p* < 0.001).

In order to probe the underlying functions of LINC01419 in HCC, we constructed lentiviral shRNA vectors and stably knockdown the endogenous expression of LINC01419 in the Huh7 and PLC/PRF/5 cells. The knockdown efficiency was confirmed by comparing with the respective controls (Figure 1(e)). The wound-healing assay showed that the expression of LINC01419 may promote HCC cell migration (Figure 1(f)). From the nucleocytoplasmic separation analysis, LINC01419 was mainly detected in the cytoplasm and partly in the nucleus (Figure 1(g)). From the CCK-8 and colony formation assays, it can be seen that LINC01419 increase the proliferative capacity Huh7 and PLC/PRF/5 cells (Figures 2(a) and 2(b)). These results suggest that high level of LINC01419 promotes HCC cell proliferation in vitro. Flow cytometry showed that after LIN01419 silencing in Huh-7 and PLI/PLF/5, the cell cycle was arrested in G0/G1 phase, and the apoptosis of HCC was significantly increased (Figures 2(c) and 2(d)).

### 3.3. LINC01419 Regulates Genome Stability

**Figure 3:**
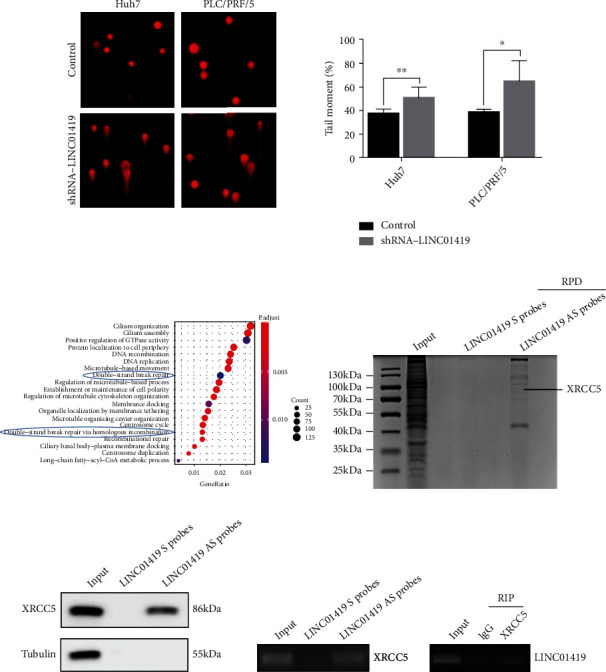
LINC01419 mediates DNA damage in HCC cells. (a) LINC01419 increases the genomic stability of the HCC cell. (b) LINC01419 was closely related to DSB damage repair from KEGG analysis. (c) The interacting proteins with LINC01419 were captured by using RNA antisense purification coupled with RAP-qMS. (d, e) LINC01419 can specifically bind the possible protein to XRCC5 (^∗^*p* < 0.05, ^∗∗^*p* < 0.01).

To further investigate the effects of LINC01419 insufficiency on genome stability, alkaline comet assay was used to detect DNA damage in HCC cells, and the results demonstrated that the formation of comet tails was significantly increased in cells depleted with LINCO1419, suggesting that this lncRNA reduces DNA damage in HCC cells. Therefore, the presence of LINC01419 increases the genomic stability of HCC cells (Figure 3(a)).

### 3.4. LINC01419 Directly Binds Ku80 and Regulates Its Phosphorylation in HCC Cells

**Figure 4:**
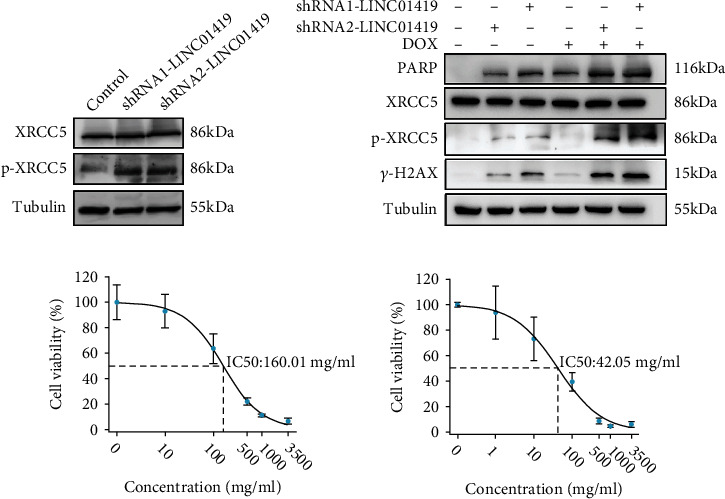
LINC01419 mediates DNA damage in HCC cells by regulating the phosphorylation level of XRCC5. (a) Phosphorylation level of XRCC5 increased with lacking of LINC01419. (b) Doxorubicin treatment is more likely to accelerate HCC death after LINC01419 deletion. (c) The sensitivity of cells to doxorubicin was significantly increased after knocking down LINC01419.

How does LINC01419 impact DNA damage? We want to identify proteins that could interact with LINC01419 in living HCC cells, and we first performed RNA sequencing in LINC01419 knockdown Huh7 cells and KEGG to analyze the possible signaling pathways involved. The results indicated that LINC01419 was closely related to DSB damage repair (Figure 3(b)). After that, we performed quantitative mass spectrometry (RAP-qMS); the results showed that LINC01419 is specifically bound to XRCC5 (Ku80) (Figures 3(c)–3(e)). Finally, we discovered that the expression of phosphorylated Ku80 increased in HCC cell with depleted LINC01419 (Figure 4(a)). Therefore, we conclude that LINC01419 mediates DNA damage in HCC cells by regulating phosphorylated XRCC5.

### 3.5. Genetic Knockdown of LINC01419 Sensitized HCC Cells to Doxorubicin

Drug-eluting bead transarterial chemoembolization (DEB-TACE) is currently widely used in advanced hepatocellular carcinoma [16]. To test the role of LINC01419 in doxorubicin resistance, we treated the Huh7 cell line with doxorubicin and observed obvious increase in the DNA damage in the LINC01419 knockdown HCC cells (Figure 4(b)). Furthermore, the results showed that after knocking down LINC01419, the sensitivity of cells to doxorubicin was significantly increased (Figure 4(c)). This suggests that loss of LINCO1419 expression makes HCC cells more sensitive to doxorubicin.

## 4. Discussion

Many researches have shown that lncRNAs are the pivotal regulators of various genes which are essential for HCC, for example, lncRNAs mainly regulate the epigenetics of liver cancer through transcription factors, DNA methylation, histone acetylation, etc. [17]. Interestingly, disturbance in lncRNAs expression may alter the immune response which can lead to chronic hepatitis, abnormal hepatocyte proliferation, and further lead to the development of HCC [18]. This further illustrates that the importance of lncRNAs and LINC01419 is a lncRNA located on human chromosome 8, which can directly bind to EZH2 to promote histone methylation of promoters, thereby promoting HCC progression [19]; it can be activated and induced by interacting with NDRG1 promoter [20]. Here, we revealed the correlation of LINC01419 with DNA damage in HCC cells, elucidating the unique role of LINC01419 in HCC from a new perspective.

In our study, we first explored the HCC data in TCGA and found that LINC01419 was upregulated in HCC and then examined the expression of LINC01419 in 41 HCC patients, which was consistent with its expression trend in the TCGA database.

Following the silencing of LINC01419 expression, HCC cells exhibited suppressed proliferation, migration, wound healing, and colony formation. We were intrigued by the increase in comet tails after deprivation of LINC01419 expression in comet experiments. DNA damage occurs in hundreds of millions of cells in the human body per day but the repair mechanisms fix it [21]. An imbalance of coordination between DNA damage and repair functions can be observed in a variety of human cancers [22]. As mentioned above, NHEJ can occur in the overall process of cell division; DNA-PK is the core substance of NHEJ, which consists of one catalytic subunit DNA-PKcs and Ku70/Ku80 [23]. Firstly, DSB is recognized by Ku protein, then binds and activates the protein kinase DNA-PKcs, and recruits and activates various catalytic enzymes required for DNA repair [24]. Then, the Ku-XRCC4-DNA ligase complex initiates the NHEJ pathway after DNA ends are recognized [5]. In the current study, we used mass spectrometry to analyze proteins that can bind to LINC01419; the XRCC5 (Ku80) caught our attention, because it is known to DNA damage repair in various cancers, and then, the direct binding of LINC01419 to XRCC5 was determined. Correspondingly, we performed an RNA sequence in LINC01419-depleted HCC cells to explore the underlying mechanism. KEGG functional enrichment of related genes downregulated after LINC01419 deletion and found to be significantly associated with DNA damage.

AS a regulatory subunit of the DNA-PK that phosphorylates many proteins, such as RNA-polymerase, P53, and many transcription factors [25]. While XRCC5 is phosphorylated or ubiquitinated when it binds to DSB, current studies have shown that XRCC5 is phosphorylated when it forms a dimer with Ku70 required for DNA repair initiation [26, 27]. When we knocked down LINC01419 in HCC cells, we found that the phosphorylation level of XRCC5 increased, indicating that LINC01419 regulates DNA damage by regulating the phosphorylation of XRCC5. Besides, all causes of chronic liver disease are associated with genomic stability, and these changes can be detected in the early stages of the disease [28]. Studies have shown that individuals with HCC are more susceptible to DNA damage, which further illustrates the role of genomic stability in the development of HCC [29]. In our research, the silencing of LINC01419 was accompanied by an increase in *γ*-H2AX, implying increased DNA damage, suggesting that the upregulated LINC01419 in HCC promotes tumor progression by maintaining genomic stability.

The most common treatment for HCC is surgical resection, but patients with advanced HCC are not suitable for surgical treatment. In this case, chemotherapy or immune-targeted therapy are the best options [30]. As the treatment progresses, some patients will develop drug resistance; thus, only a few patients could effectively be treated. The doxorubicin (Dox) is the cornerstone of HCC chemotherapy, a commonly used drug for trans-arterial chemoembolization (TACE) in unresectable HCC [31, 32]. Our findings confirm that LINC01419 increases HCC's resistance to Dox.

In summary, in the present study, we elucidated the role of LINC01419 in HCC development; thus, we suggest LINC01419 as a potential therapeutic target for HCC. Interestingly, we are for the first time elaborating on the role of LINC01419 in Dox resistance in HCC, which provides a new theoretical basis for future research on Dox resistance in HCC.

## Figures and Tables

**Table 1:** Primer sequences used in this study.

Primer	Sequences (5′-3′)
LINC01419(F)	AGCCAAACCTAATAAAACCAGC
LINC01419(R)	ACAGTCTCCCCTTTGTGATTT
shRNA1-LINC01419(F)	CCGGGGAATTCTCCCAAATGTATGGATCCATACATTTGGGAGAATTCCTTTTTTG
shRNA1-LINC01419(R)	AATTCAAAAAAGGAATTCTCCCAAATGTATGGATCCATACATTTGGGAGAATTCC
shRNA2-LINC01419(F)	CCGGCTATGAAGCCAAACCTAATGGATCCATTAGGTTTGGCTTCATAGTTTTTTG
shRNA2-LINC01419(R)	AATTCAAAAAACTATGAAGCCAAACCTAATGGATCCATTAGGTTTGGCTTCATAG
GAPDH(F)	ATGTTGCAACCGGGAAGGAA
GAPDH(R)	CGCCCAATACGACCAAATCAGA

**Table 2:** Correlation between the expression of LINC01419 and the clinicopathological characteristics of HCC patients.

Clinicopathologic characteristics	*n*	LINC01419		*p* value
		High expression	Low expression	
Age (year)				0.923
≤60	16	6	10	
>60	25	9	16	
Gender				0.975
Male	19	7	12	
Female	22	8	14	
Viral status				0.273
Positive	35	14	21	
Negative	6	1	5	
Serum AFP (ng/mL)				0.460
≤20	6	3	3	
>20	35	12	23	
Liver cirrhosis				0.613
Yes	37	14	23	
No	4	1	3	
Tumor size (cm)				0.020
≤5 cm	18	3	15	
>5 cm	23	12	11	
Thrombus				0.001
Positive	12	9	3	
Negative	29	6	23	
Micro vascular invasion (MVI)				0.024
Yes	13	8	5	
No	28	7	21	
TNM stage				0.013
I-II	24	5	19	
III-IV	17	10	7	
Tumor differentiation				0.026
Well	8	1	7	
Moderate	25	8	17	
Poor	8	6	2	
Lymph nodes metastasis				0.024
Yes	13	8	5	
No	28	7	21	
Recurrence				0.608
Yes	17	7	10	
No	24	8	16	

## Data Availability

The data generated during the study could be requested from the corresponding authors on suitable requirements.
